# Insecticide resistance and genetic structure of *Aedes aegypti* populations from Rio de Janeiro State, Brazil

**DOI:** 10.1371/journal.pntd.0008492

**Published:** 2021-02-16

**Authors:** Rafi Ur Rahman, Luciano Veiga Cosme, Monique Melo Costa, Luana Carrara, José Bento Pereira Lima, Ademir Jesus Martins

**Affiliations:** 1 Laboratório de Fisiologia e Controle de Artrópodes Vetores, Instituto Oswaldo Cruz/ FIOCRUZ, Rio de Janeiro, Brazil; 2 Department of Ecology and Evolutionary Biology, Yale University, New Haven, United States of America; 3 Instituto Nacional de Ciência e Tecnologia em Entomologia Molecular, Rio de Janeiro, Brazil; University of Cincinnati, UNITED STATES

## Abstract

Vector control largely relies on neurotoxic chemicals, and insecticide resistance (IR) directly threatens their effectiveness. In some cases, specific alleles cause IR, and knowledge of the genetic diversity and gene flow among mosquito populations is crucial to track their arrival, rise, and spread. Here we evaluated *Aedes aegypti* populations’ susceptibility status, collected in 2016 from six different municipalities of Rio de Janeiro state (RJ), to temephos, pyriproxyfen, malathion, and deltamethrin. We collected eggs of *Ae*. *aegypti* in Campos dos Goytacazes (Cgy), Itaperuna (Ipn), Iguaba Grande (Igg), Itaboraí (Ibr), Mangaratiba (Mgr), and Vassouras (Vsr). We followed the World Health Organization (WHO) guidelines and investigated the degree of susceptibility/resistance of mosquitoes to these insecticides. We used the Rockefeller strain as a susceptible positive control. We genotyped the V1016I and F1534C knockdown resistance (*kdr*) alleles using qPCR TaqMan SNP genotyping assay. Besides, with the use of *Ae*. *aegypti* SNP-chip, we performed genomic population analyses by genotyping more than 15,000 biallelic SNPs in mosquitoes from each population. We added previous data from populations from other countries to evaluate the ancestry of RJ populations.

All RJ *Ae*. *aegypti* populations were susceptible to pyriproxyfen and malathion and highly resistant to deltamethrin. The resistance ratios for temephos was below 3,0 in Cgy, Ibr, and Igg populations, representing the lowest rates since IR monitoring started in this Brazilian region. We found the *kdr* alleles in high frequencies in all populations, partially justifying the observed resistance to pyrethroid. Population genetics analysis showed that *Ae*. *aegypti* revealed potential higher migration among some RJ localities and low genetic structure for most of them. Future population genetic studies, together with IR data in *Ae aegypti* on a broader scale, can help us predict the gene flow within and among the Brazilian States, allowing us to track the dynamics of arrival and changes in the frequency of IR alleles, and providing critical information to improving vector control program.

## Author summary

Monitoring of insecticide resistance (IR) is mandatory for the effectiveness of vector control programs. We investigate six populations of *Aedes aegypti* regarding their IR status towards insecticides previously or currently in use, their frequency of IR alleles and resistance ratio, their genetic ancestry and structure, and the gene flow. We observed that *Ae*. *aegypti* populations from RJ are becoming susceptible to the larvicide organophosphate temephos. The resistance ratios of temephos are lower among populations where gene flow might be occurring. All RJ populations are highly resistant to deltamethrin, partially explained by the high frequency of *kdr* alleles. Besides, all populations are susceptible to pyriproxyfen’s insect growth regulator and the organophosphate malathion. Population genomic analyses using more than 15,000 SNPs identified three potential genetic clusters within RJ populations, with higher gene flow to and out of the touristic region called Região dos Lagos. IR data, together with knowledge of the genetic structure and gene flow among different mosquito populations, can help with vector control program decisions.

## Introduction

In 2016, Brazil’s number of dengue cases was near 1.5 million, representing 69% of the dengue cases in South, Central, and North America combined. Within the Southeast region of Brazil, Rio de Janeiro State (RJ) is a crucial hub for the import and export of goods and a tourist attraction and port of entry. In 2016, RJ registered high dengue, chikungunya, and Zika, with 443.7, 108.1, and 414.2 cases per 100,000 inhabitants, respectively [1]. The State capital Rio de Janeiro City (Rio) and the Região dos Lagos are the leading destinations for domestic and foreign visitors. The region also plays a significant role in introducing mosquitoes, mostly in eggs attached to goods that travel by road, air, sea, and arboviruses affecting humans. Historical records show most of the dengue virus emergencies from Brazil in RJ [2–5].

*Aedes aegypti* is the vector of dengue virus (DENV), chikungunya virus (CHIKV), yellow fever virus (YFV), and Zika virus (ZIKV). Most RJ urban centers have ideal climatic and breeding conditions to maintain the mosquitoes under high densities, especially during warmer seasons [6,7].

Improvements in urban sanitary infrastructure are necessary to avoid the accumulation of water and discard wastes appropriately, significantly reducing the artificial breeding sites ideal for the early development of *Ae*. *aegypti* larvae. However, this is a complicated task, especially in the underdeveloped tropical countries with densely populated cities in complex urban landscapes, as RJ. The deployment of insecticides has been playing an essential role in vector control. However, their intense use resulted in insecticide resistance in *Ae*. *aegypti* populations around the world, affecting vector control on global scales [8]. This is particularly true for the organophosphate (OP) larvicide temephos and the adulticide pyrethroids (PY) deltamethrin [9]. As a result of resistance development against temephos and deltamethrin, the Brazilian Ministry of Health (MoH) replaced the larvicide with insect growth regulators (IGRs) and the adulticide with malathion [10,11].

*Ae*. *aegypti* response to neurotoxic insecticides may be through physiological resistance or modifications in behavior. Among the physiological resistance mechanisms, enzymatic detoxification of insecticides (metabolic resistance) and modifications in the target site (target site resistance) are very well documented [12]. Target site modifications are single nucleotide polymorphisms (SNPs) in the voltage-gated sodium channel (*Na*_*V*_), known to confer resistance to the pyrethroids’ knockdown effect, and are, therefore, named *kdr* mutations [13]. At least two *kdr* alleles are present in *Ae*. *aegypti* from Brazil: Na_V_R1, with the substitution F1534C, and Na_V_R2, with both F1534C and V1016I. We observed two *kdr* alleles at high frequencies in pyrethroid-resistant *Ae*. *aegypti* populations from RJ [14]. A third SNP in the same gene (IS6 segment), V410L in the Na_V_R2 allele, raises concerns, increasing the resistance magnitude [15–17].

In Brazil, there are two main distinct genetic clusters of *Ae*. *aegypti* with a break separating Northern and Southern populations, possibly originating from two independent re-invasions. The Northern group is genetically close to Venezuelan populations, whereas the Southern cluster is genetically close to Dominica and the Caribbean [18,19]. Few studies have provided insights into the genetic structure of *Ae*. *aegypti* in RJ. A previous study using six isoenzyme loci looked at the geographic and temporal generic patterns of *Ae*. *aegypti* in the city of Rio de Janeiro between 2002 and 2003 [20], finding relatively high genetic differentiation. However, a recent study using twelve microsatellite markers showed that RJ mosquitoes display high levels of admixed ancestry and genetic variability [18].

To elucidate the physiological mechanisms and provide clues about the evolution of insecticide resistance (IR) and cross-resistance among different classes of insecticides in natural vector populations, extensive analysis of insecticide resistance are essential [21]. Wild populations’ genetic structure may help determine the gene flow and provide critical information about insecticide resistance alleles dissemination [22,23]. Here we evaluated the resistance status of previously used and in-practice insecticide in the control of *Ae*. *aegypti* populations from representative regions of RJ and the frequencies of *kdr* mutations are among the primary mechanisms selected for pyrethroid resistance. Besides, we used a new molecular tool to determine these populations’ genetic structure, based on a population genomics approach. With these data, we were able to assess the genetic diversity and differentiation of *Ae*. *aegypti* in RJ and to evaluate the interplay of genetic structure and IR levels throughout the RJ state.

## Methods

### Ethics statement

In this study, we used guinea pigs anesthetized with xylazine and ketamine, as the blood-feeding source for female mosquitoes. This was approved by the *Comissão de Ética no Uso de Animais do Instituto Oswaldo Cruz*, (CEUA/IOC), under the license number 003/2018.

### Characterization of the sampled localities

**Fig 1:**
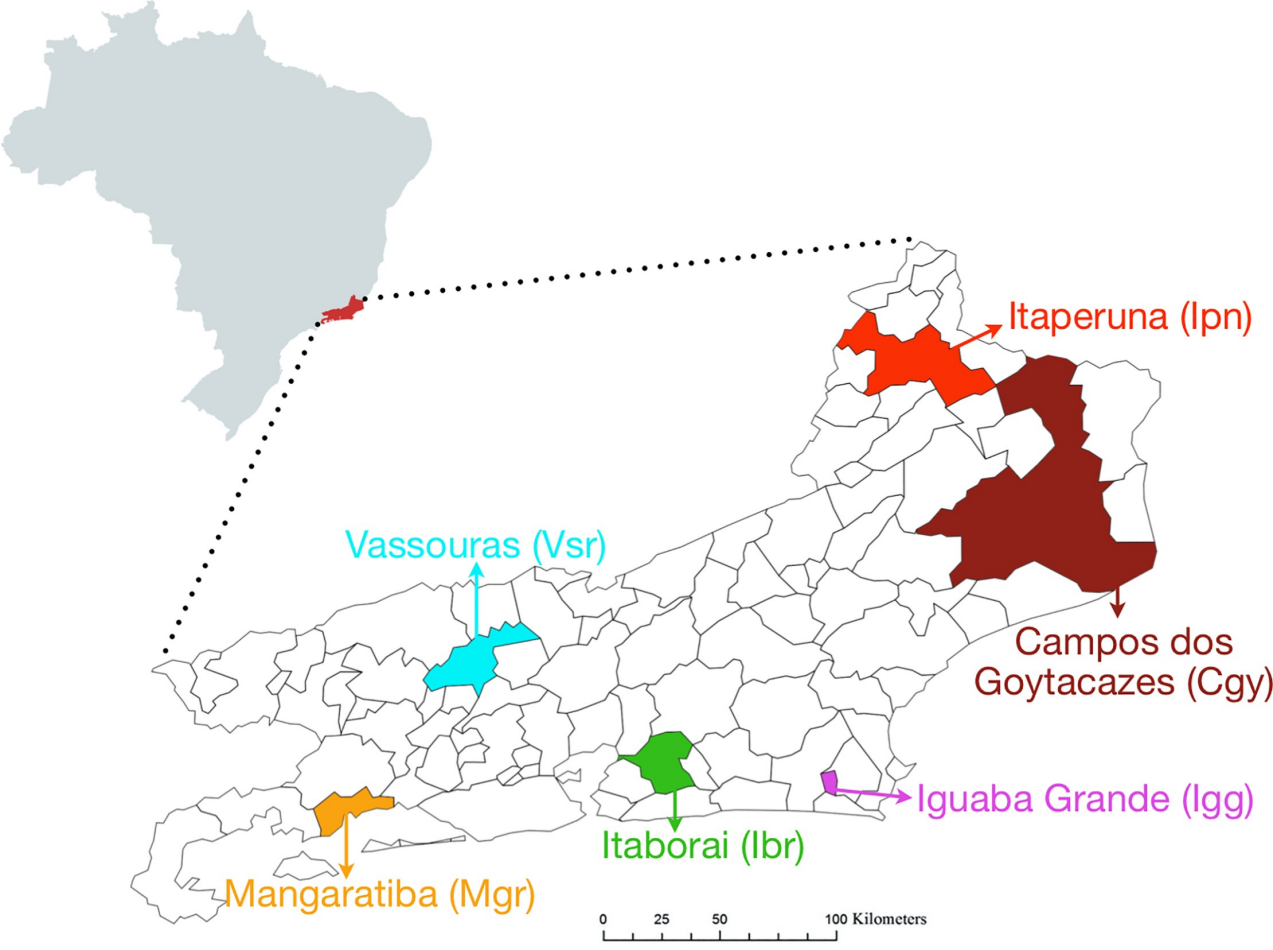
Map of Rio de Janeiro State with the six municipalities where we collected *Aedes* spp. for our study. (Maps were created with open-source scripts in R platform. The shapefiles used to insert the correct map boundaries were all retrieved from public domain sources: Geobr R package, available at https://cran.r-project.org/web/packages/geobr/vignettes/intro_to_geobr.html).

According to the Brazilian Institute of Geography and Statistics, Rio de Janeiro State (RJ) is the second most populated Brazilian State after São Paulo and was the fourth in the human developmental index, according to the 2010 census [24]. We chose representative municipalities of the principal geographic/administrative regions of the RJ state to explore the insecticide effectiveness in *Ae*. *aegypti*, their genetic structure and gene flow among them (Fig 1): Campos dos Goytacazes (Cgy) in the North, Itaperuna (Ipn) in Northwest, Iguaba Grande (Igg) in the Coastal Lowland (Região dos Lagos), Itaboraí (Ibr) in the Metropolitan Region II, Mangaratiba (Mgr) around the Ilha Grande bay and Vassouras (Vsr) in the Center-South region (Fig 1). The geographic details about these localities are in Table 1. Among these cities, Ibr is the closest to the Rio de Janeiro city; Mgr to the south and Igg to the north are touristic hotspots; Vsr, Ipn, and Cgy have significant agricultural production in their rural territories.

**Table 1:** The localities of sampling eggs of *Aedes* spp. for this study and their geographical parameters.

Localities	Code	Geographic coordinates	Population ^a^	Area (km^2^)	Demographic density ^b^	With sanitary sewer ^c^
Campos dos Goytacazes	Cgy	21°45′14″S, 41°19′26″W	487,186	4,031.99	120.83	57.1%
Itaperuna	Ipn	21°12’18″S, 41°53’16″W	99,504	1,106.17	89.95	88.1%
Iguaba Grande	Igg	22°50′20″S, 42°13′44″W	26,430	50.54	522.92	85.8%
Itaboraí	Ibr	22°44′38″S, 42°51′32″W	230,786	430.44	536.17	65.2%
Vassouras	Vsr	22°24′14″S, 43°39′46″W	35,622	535.64	66.50	77.0%
Mangaratiba	Mgr	22°57′36″S, 44°02′27″W	41,557	358.56	115.90	73.5%
RJ State	RJ		16,635,996	43,750.42	380.25	

a Number of inhabitants estimated for 2016. Source: [Instituto Brasileiro de Geografia e Estatística]—IBGE

b Inhabitants per Km^2^

c Houses with adequate sanitary sewage system in 2018. Source: [Instituto Brasileiro de Geografia e Estatística]–IBGE

### Collections of eggs and maintenance of colonies in the laboratory

We received eggs from the municipalities, as mentioned earlier, collected using ovitraps following the protocol of the Brazilian National Program of Dengue Control (PNCD) with some modifications [11]. We collected eggs from 100–120 ovitraps placed near the houses in each locality, covering all the locality’s urban areas. Each trap consisted of a black plastic cup with 300 mL water and 1 mL of yeast extract solution at a final concentration of 0.04%. We used wood paddles to collect eggs for two weeks, every 5^th^ or 7^th^ day, then left them to dry in the air for a couple of days and transported them to the laboratory. In the insectary, we mixed all the paddles containing eggs and stimulated the eggs to hatch in cups containing 1 L of dechlorinated water. After three days, we transferred all the larvae to new trays, each containing 300 larvae in 2 L dechlorinated water. We fed the larvae with fish food (Tetra-Marine Granules, Tetra) in a room maintained at 28 ± 2°C until they reached the pupal stage.

We transferred the pupae to labeled cages, and once the adults emerged, we identified them to species level (*Aedes aegypti* and *Aedes albopictus*) using known morphological characteristics [25]. We put adults in new labeled cages and fed them with 10% sugar solution *ad libitum* in an insectarium under 25 ± 2°C temperature and relative humidity 75 ± 10%, in a regimen of 12/12 hours light/ dark period.

After 3–4 days, we kept the male mosquitoes for molecular analysis at -20°C. We used guinea pigs, anesthetized with xylazine and ketamine, according to license CEUA/IOC-003/2018, to feed the females, which we starved for 12h. We put black oviposition cups internally covered with white filter paper in each cage for the oviposition and obtained eggs after three days. We preferably used F1 for bioassays, but we used F1 to get new generations if the egg yield was low. Laboratory susceptible strain, Rockefeller (to be called Rock from now on), was used to reference insecticide susceptibility and standard laboratory vigor in all tests [26].

### Larval bioassays

#### Temephos

We performed dose-response tests with the organophosphate temephos (Pestanal Sigma-Aldrich), following the WHO guideline procedures [27]. We used ten concentrations (0.0009–0.066 mg/L), each in four replicates and repeated 3–5 times. Each replica means a disposable cup of 150 mL capacity, containing 100 mL solution with ~20 third instar (L3) larvae. The negative control consisted of four replica cups containing the solvent instead of insecticide. We recorded mortality after 24h of exposition. We applied Abbott’s correction for the calculation of control mortality if necessary [28]. Using *probit* analysis, we calculated the lethal concentration (LC) and, subsequently, resistance ratio (RR), which is the ratio between the LC of each population and Rock [29,30]. Another critical parameter to consider in *probit* analysis is the slope, the more heterogeneous a population is, the smallest is its slope and vice versa. We maintained a regime of temperature, relative humidity, and light cycle as 25°C, 75 ± 10%, and 12/12h light/dark.

#### Pyriproxifen

For this IGR, we performed a dose-diagnostic assay [31]. Each assay contained 14 treatments with pyriproxyfen (Sigma-Aldrich) at 0.18 mg/L and six control treatments (containing solvent instead of insecticide). Each treatment means a 250 mL solution in a disposable, transparent plastic cup of 300 mL capacity. We added ten L4 larvae and fish food (Tetra-Marine Granules, Tetra) and covered it with netting to avoid eventual adult escaping to each cup. We recorded mortality and life stage changes daily. We used the Rock strain in parallel to the susceptibility control. The emergence of all larvae into adults in the cups without the IGR indicated the assay’s completion. We repeated the assays three times under the same environmental conditions as described above for temephos tests. Unlike neurotoxic insecticides, we do not evaluate mortality levels of IGRs themselves but the index of Adult Emergency Inhibition (AEI). We considered a population resistant when AEI is less than 90%. We also evaluated the proportions of mortality at each stage: larvae, pupae, and unsuccessfully emerged adults (adults that remained attached to the exuviae and died).

### Adult bioassays

#### Deltamethrin

We followed the WHO qualitative tube test procedure for the pyrethroid deltamethrin with few modifications [32,33]. We impregnated papers on our own with technical grade deltamethrin (Pestanal Sigma-Aldrich). We dissolved deltamethrin in acetone to a 10% stock concentration and then diluted it with silicone oil (Dow Corning) to the 0.05% concentration. We then applied 840 μL to 12 X 15 cm filter papers (Whatman grade 1). In each assay, 20 female mosquitoes, 3–4 days old, non-blood-fed, in a resting tube (tube without insecticide). We exposed these mosquitoes to the diagnostic dose of insecticide for 1 hour, during which we counted *knockdown* mosquitoes every 5 minutes. We transferred the mosquitoes back to the resting tube, and after 24 hours, we recorded the mortality.

#### Malathion

For the organophosphate malathion, we adopted the CDC bottle assays with a diagnostic concentration [34]. We impregnated 250 mL glass bottles (Wheaton) with 1 mL of 20 μg/mL malathion (Cheminova Brasil Ltda, São Paulo) dissolved in acetone. We exposed around 25 mosquitoes to the insecticide for one hour, with mortality recorded every 15 minutes in each assay.

We conducted assays with four replicas for both adulticides (deltamethrin and malathion). We then repeated the assay three times for each population, using positive (Rock) and negative (population exposed to the solvent) controls. We kept the adults in constant temperature, relative humidity, and the light cycle at 25 ± 2°C, 75 ± 10%, and 12/12h light/dark, respectively. Following the WHO criteria, we considered populations resistant when mortality was lower than 90% [31].

### *Kdr* Genotyping

We performed the *kdr* genotyping for 43–46 mosquitoes to quantify the known alterations Val1016Ile and Phe1534Cys in the voltage-gated sodium channel gene (*Na*_*V*_). We first extracted genomic DNA with *NuleoSpin DNA insect kit* (Macherey-Nagel Laboratories), following manufacturer’s instructions, eluted it in 30 μL ultra-pure water, quantified its concentration with NanoDrop One (ThermoFisher Scientific), and diluted it to 20 ng/μL for a customized qPCR TaqMan SNP genotyping approach.

Each genotyping reaction contained: 1X *TaqMan Genotyping Master Mix* (Thermo Fisher), 1X *Custom TaqMan SNP Genotyping Assay* (ThermoFisher, AHS1DL6 for V1016I or AHUADFA for F1534C), 20 ng DNA, and 10 μL ultra-pure water. We used a thermocycling program according to the manufacturer’s instruction and endpoint reading at the 40^th^ cycle in QuantStudio 6 qPCR equipment (ThermoFisher).

We used the DNA of Rock (SS) and Rock-*kdr* (RR) strains as positive controls for wild-type homozygous and *kdr* homozygous, respectively. We mixed equal amounts of both Rock and Rock-*kdr* strains’ DNA as a control for heterozygous alleles (SR) [35].

We genotyped mosquitoes for 1016 (Val^+^ or Ile^*kdr*^) and 1534 (Phe^+^ or Cys^*kdr*^) SNPs, with primers and probes previously described [36]. Considering both sites were present on the same locus, we are aware of three alleles in Brazilian *Ae*. *aegypti* populations: the wild-type *Na*_*V*_*S* (V1016 + F1534) and the *kdr Na*_*V*_*R1* (V1016 + 1534C) and *Na*_*V*_*R2* (1016I + 1534C), combining in six possible genotypes (SS, SR1, SR2, R1R1, R1R2, and R2R2). We calculated frequencies (both allelic and genotypic) and the Hardy-Weinberg Equilibrium test for each population [37].

### Population genomics

#### SNP genotyping

We extracted whole genomic DNA from mosquito samples using the QIAGEN Blood and Tissue kit (Qiagen, Germany) following the manufacturer’s instructions. For genotyping using an SNP microarray for *Ae*. *aegypti*, we submitted 12 genomic DNA samples from each population to the Functional Genomics Core at the University of North Carolina, Chapel Hill. This SNP-chip was developed with ddRAD data from *Ae*. *aegypti* populations around the world. There are 50,000 polymorphic sites represented on the chip. It can be used for fine demographic studies, genetic structure, etc. A detailed description is found elsewhere [38]. Although the SNP-chip was developed with previous versions of *Ae*. *aegypti* genome, all the probes have been mapped to the AaegL5 genome available on Vectorbase.org.

We processed the data in the Axiom Analysis Suite v.3.1 (Affymetrix, Santa Clara, CA) to generate the genotype calling. We performed genotype calling separately for each analysis that includes different sets of samples. Next, we filtered the SNP data in PLINK v 1.9 [39,40] to remove: 1) loci with more than 10% missingness, 2) individuals with more than 10% missing loci, 3) loci that failed Hardy-Weinberg tests with a threshold of 0.00001 for each population [41], 4) loci with a minor allele frequency smaller than 5%, and 5) samples whose expected heterozygosity values deviate more than ±3 standard deviations from the mean of all samples, which might indicate low DNA quality, contamination or high inbreeding [42]. This study employed the term "population" to differentiate mosquitoes collected in different cities, despite their genetic relationships. We did not know the genetic structure and how many populations are present in RJ. We, from now on, call the samples collected in each city as "population".

#### Inferring genetic ancestry

We examined the genetic origin of *Ae*. *aegypti* RJ populations using clustering analysis with the R package LEA by estimating each ancestry coefficient [43]. We included eleven to twelve individuals per population and an SNP dataset of 15,321 loci after quality control filtering for this analysis. We performed ten replicates for each K from 1 to 6. The best K was determined by having the lowest cross-entropy. We also used SNP data from populations previously published [44] to perform the same clustering analyzes (S1 Table). We used the python package “pong” [45] to generate the structure like plots considering all runs from LEA.

#### Population structure

We applied principal component analysis (PCA) in LEA [43] to summarize the genetic variability in our samples and visualize the genetic distances between individuals. Besides, we performed Discriminant Analysis of Principal Components (DAPC) [46] on the allele frequencies of the same groups we used in LEA and plotted the results using the R package Adegenet [47]. We estimated the pairwise genetic distance (*F*_*st*_) using the R package StTAMPP [48]. We calculated the *F*_*st*_ values across each locus based on allele frequency and the level of heterozygosity, according to Weir & Cockerham [49], taking into account the population size. We used 100 bootstraps to estimate *p*-values and confidence intervals. Lastly, we constructed a phylogeny that consisted of our populations with *Aedes mascarensis* as the outgroup, using 16,596 SNPs in IQTree v 1.6.12 [50]. IQTree conducts automatic model selection (option “MFP”) [51], ascertainment bias adjustment (option “ASC”) [52], and model violation check (option “bnni”). We performed ultrafast bootstraps (n = 1000) to estimate node certainty [53] and used FigTree [54] to manipulate the aesthetics of the best tree found by IQTree.

#### Detecting heterogeneous differentiation across the genome

In addition to estimating pairwise *F*_*st*_ values for all populations using the entire SNP dataset, we also estimated *F*_*st*_ values using sliding windows across the genome. We performed this analysis because aggregating all the makers to calculate a single value of *F*_*st*_ would possibly overlook genetic differentiation that occurs in specific genomic regions. We implemented this sliding-window analysis in VCFtools v 0.1.14 [55] with a window size of 1,000,000 bp and a sliding step of 10,000 bp.

#### Contemporary migration

We used BA3-SNPs [56] to estimate each population’s contemporary migration within the RJ. This software estimates the current migration rates between populations using Markov Chain Monte Carlo (MCMC) and required user-specified mixing parameters for migration rates, allele frequencies, and inbreeding coefficients. We used BA3-SNPs-autotune, provided by the authors of BA3-SNPs, to search for the ideal parameters. We did the autotuning with 300,000 interaction and burn-in of 10,000 and obtained the recommended the parameters dM = 0.2125, dA = 0.55, dF = 0.0125, for all 6 populations, using 15,321 SNP loci. Next, we run BA3-SNPs with the recommended parameters with 10,000,000 interactions and burn-in of 1,000,000. We parsed the results and plotted the first and second-generation migrants using the R package *circlize* [57].

## Results

### Epidemiological analyses

The Brazilian Ministry of Health (MoH) considers an outbreak when the incidence of Dengue cases (number of cases/ 100,000 inhabitants) exceeds 0.3%. Based on this threshold, all the localities from Rio de Janeiro State (RJ) reached epidemic status between 2015 and 2018. The only two exceptions are Cgy (2017), Igg (2017), and Mgr (2017 and 2018). The highest average dengue incidence for this period occurred in Ipn (17.6%), 6.5 greater than the RJ average (2.7%). In the year of the collection (2016), Ipn presented a dengue incidence of 37.9%. Chikungunya and Zika had the highest average incidence in Ibr, 7.2, and 4.7%, respectively (S1 Text).

### Collections

We received a total of 910 wood paddles from the six evaluated localities, with positivity percentage (paddles with attached eggs/total paddles multiplied by 100) ranging from 12.8% (Ipn) to 31% (Igg). *Ae*. *aegypti* was more abundant than *Ae*. *albopictus*, varying from 52% (Vsr) to 100% (Cgy) (Table 2).

**Table 2:** Paddles’ positivity rate and percent of *Aedes aegypti* and *Aedes albopictus* adults. Paddles’ positivity percentage means the paddles with *Aedes* spp. eggs attached to it, out of total paddles received by the lab. The last column shows the percentage of adults of each species, *Ae*. *aegypti* and *Ae*. *albopictus*. We screened adults to their species based on morphological characters soon after they emerged.

Name of Municipality	Code	Paddles			Percentage of species (%)	
		Total received	Positive	Percent positive (%)	*Ae*. *aegypti*	*Ae*. *albopictus*
Campos dos Goytacazes	Cgy	209	33	15.8	100	0
Iguaba Grande	Igg	145	45	31.0	94.7	5.2
Itabotraí	Ibr	103	30	29.1	70.5	29.4
Itaperuna	Ipn	187	24	12.8	69.4	30.3
Mangaratiba	Mgr	89	23	25.8	94.7	5.3
Vassouras	Vsr	177	26	14.7	52.3	47.6

### Insecticide resistance bioassays

#### Bioassays with larvae

We determined the level of resistance of L3 larvae to the organophosphate temephos by doing quantitative dose-response tests (Table 3).

**Table 3:** Temephos resistance levels of *Aedes aegypti* populations from Rio de Janeiro. L_C_ = lethal concentration in mg/L, values in parenthesis indicate 95% confidence interval (CI95%); RR = Resistance Ratio.

Population/ line	LC_50_		LC_90_		slope	RR_50_	RR_90_
Rockefeller	0.004	(0.002–0.006)	0.009	(0.006–0.012)	4.07	-	-
Cgy	0.012	(0.008–0.016)	0.022	(0.015–0.032)	4.65	2.8	2.6
Ibr	0.012	(0.009–0.017)	0.026	(0.019–0.034)	3.80	2.9	3.0
Igg	0.010	(0.007–0.013)	0.022	(0.015–0.032)	3.63	2.3	2.5
Ipn	0.027	(0.017–0.040)	0.061	(0.034–0.106)	3.55	6.3	7.1
Mgr	0.014	(0.011–0.018)	0.027	(0.021–0.036)	4.40	3.3	3.2

**Fig 2:**
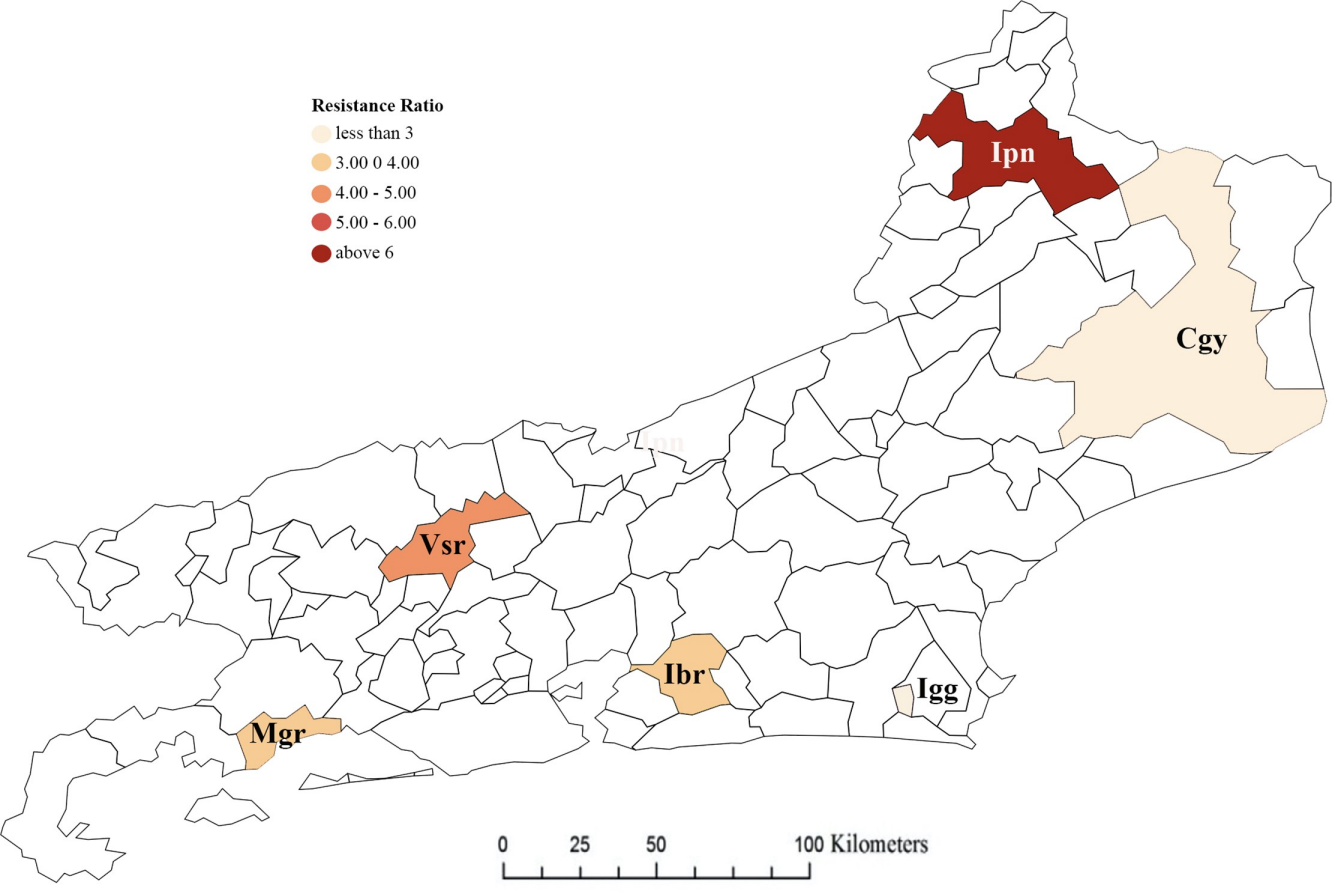
Resistance ratios (RR_50_) to temephos in *Aedes aegypti* populations from Rio de Janeiro State, Brazil. We obtained the RR_50_ after calculating the lethal concentration (LC_50_) of each population and comparing it with the LC_50_ of the reference strain.

The concentration required to kill 50% of the susceptible strain (Rock) in 24h exposition, the LC_50_, was 0.004 mg/L, and for field populations, ranging from 0.010 to 0.027 mg/L. Resistance ratios (RR_50_) varied from 2.3 (Igg) to 6.3 (Ipn), indicating resistance to temephos, however, under low levels. Roughly, the populations from coastal cities were less resistant than those from the inner land, as suggested in Fig 2. The slope of the *probit* shows that Ipn is the most heterogeneous population [slope value = 3.55] while Cgy is the least one [slope value = 4.65]

**Fig 3:**
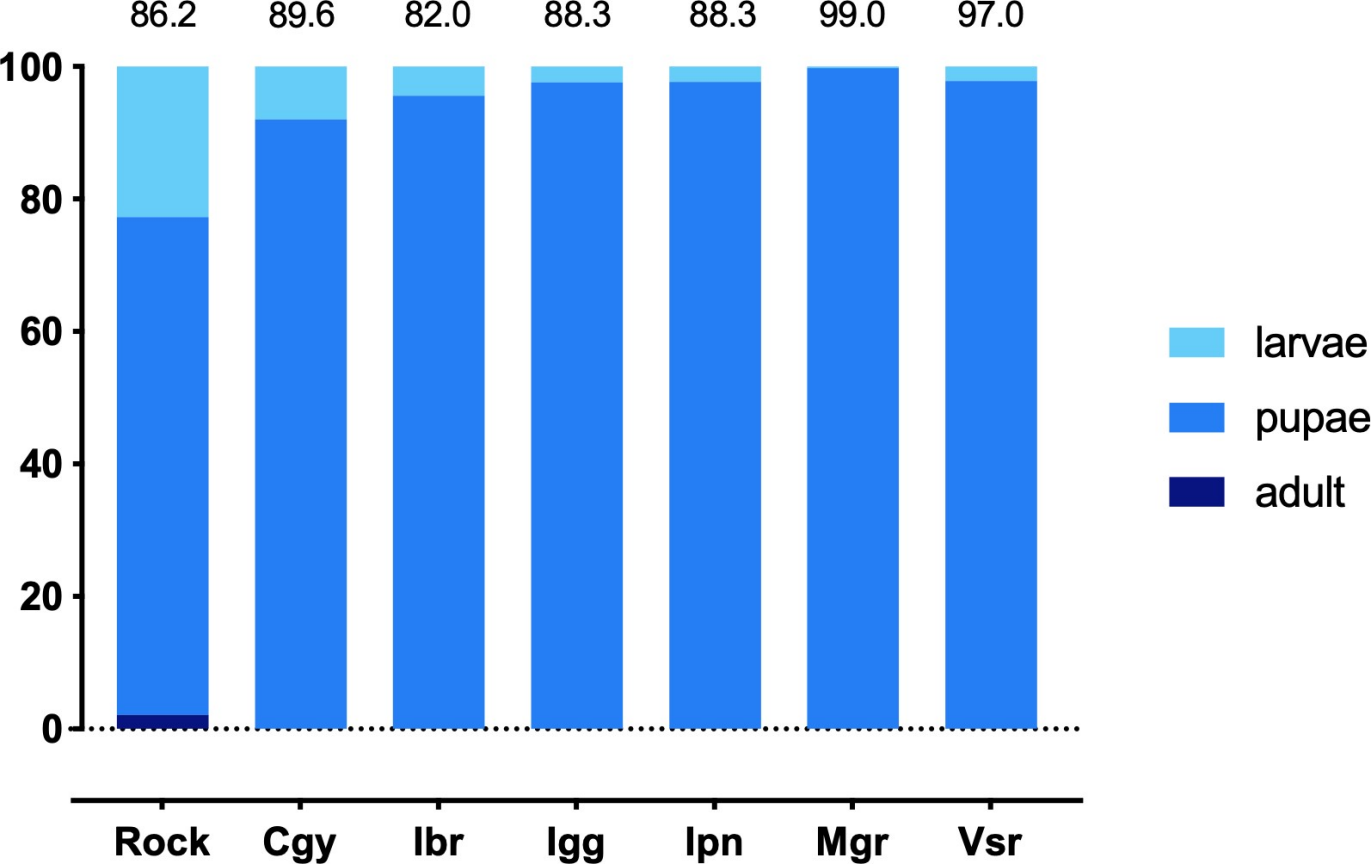
Proportional life-stage mortality of larvae of *Aedes aegypti* populations from Rio de Janeiro exposed to pyriproxyfen for 7–10 days. Adults’ emergence was 100% inhibited in the reference lineage Rockefeller (Rock) and all populations. Values over the bars indicate the mortality percentage at the end of the test (when 100% of the control condition larvae emerged into adults).

We evaluated the populations’ susceptibility status against the insect growth regulator (IGR) pyriproxyfen in a qualitative bioassay with the diagnostic dose of 0.18 mg/L. The experiment took 7 to 10 days, and none of the individuals died in the control treatment. At these time points, mortality in the treatments was between 82 and 99% (Fig 3). Rock exhibited a higher proportion of total mortality (29.4%) than some natural populations (0.21% in Mgr to 12.0% in Cgy). Pyriproxyfen caused 100% of adult emergence inhibition (AEI) in Rock and all RJ populations.

#### Bioassays with adults

**Fig 4:**
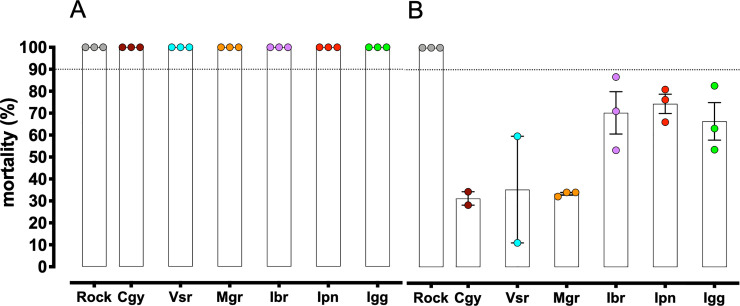
Mortality of *Aedes aegypti* from RJ populations exposed to the adulticides: (A) organophosphate malathion (20μg/bottle) and (B) pyrethroid deltamethrin 0.05%. We used Rockefeller (Rock) as the susceptible reference. Dots represent the mean mortality of each performed bioassay with total mean and SEM error bars.

A qualitative dose-diagnostic assay with the OP malathion (20 μg/mL) using the CDC bottle guidelines showed all populations are ’susceptible’ to this OP. Rock and all natural populations achieved 100% mortality during 1h of exposure (Fig 4A). WHO-tube tests using papers impregnated with 0.05% deltamethrin showed all populations resistant to this pyrethroid. Populations Cgy, Vsr, and Mgr presented mean mortalities between 31.4% and 35.4%, whereas, Ibr, Ipn, and Igg showed higher mortality, between 66.5% and 74.5% (Fig 4B).

### *Kdr* genotyping

**Fig 5:**
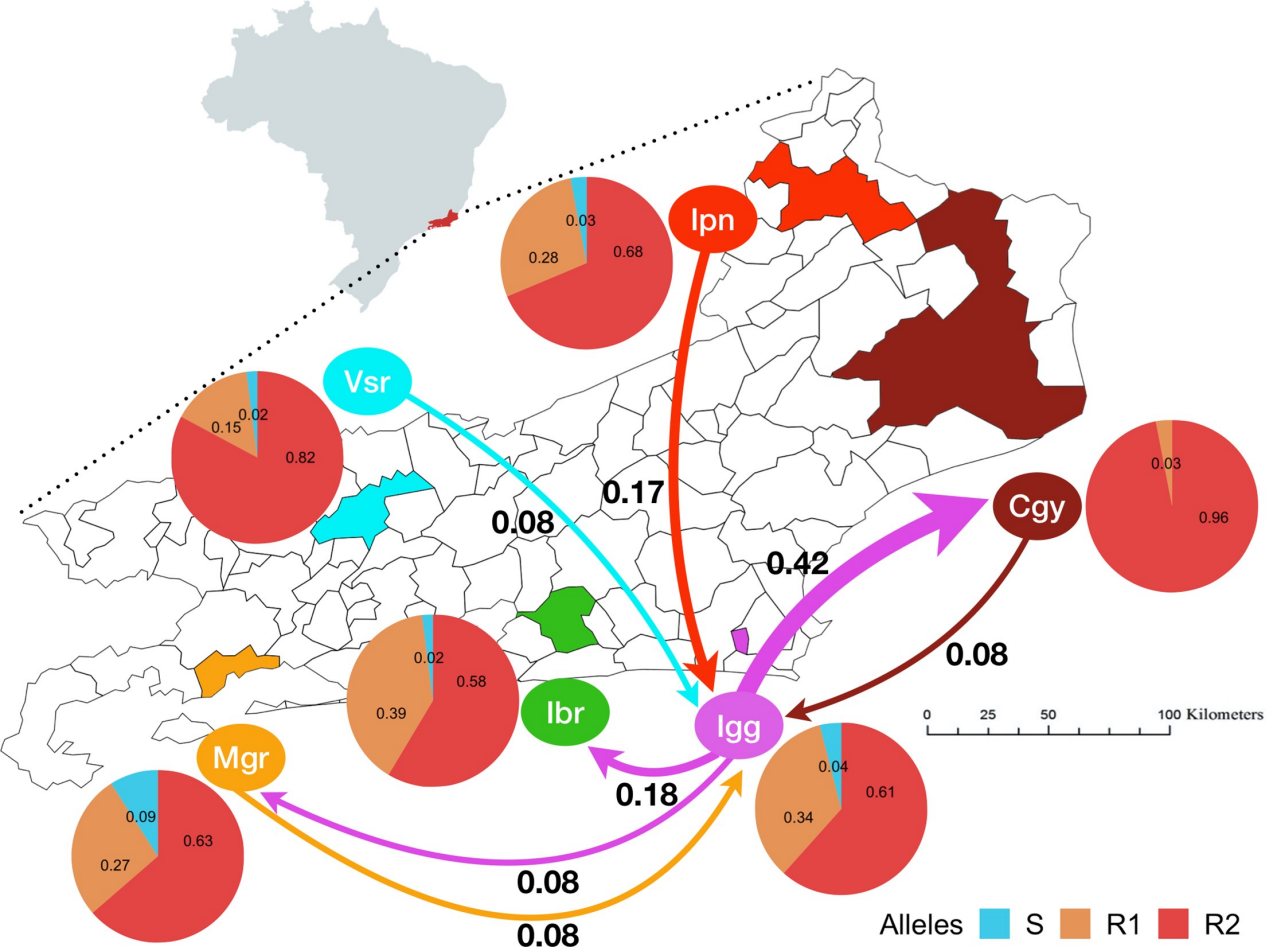
Contemporary migration estimates of *Aedes aegypti* in Rio de Janeiro State and frequency of the *kdr* alleles. The values do not indicate migration rates but are indicative of possible migration routes.

We evaluated the frequencies of *kdr* alleles in *Ae*. *aegypti* populations from RJ since they were resistant to deltamethrin. We genotyped 266 samples for two Na_V_ SNPs: 1016 (Val or Ile^*kdr*^) and 1534 (Phe or Cys^*kdr*^) (Fig 5). The Na_V_S haplotype (1016Val + 1534Phe) was found under very low frequencies, from absent in Cgy up to 9% in Ipn. The *kdr* Na_V_R1 (1016Val + 1534Cys^*kdr*^) varied between 3% (Cgy) to 34% (Mgr), while Na_V_R2 (1016Ile^*kdr*^ + 1534Cys^*kdr*^) was the most frequent haplotype, with 58% in Vsr and 96% in Cgy. The sum of the "resistant" genotypes (R1R1, R1R2, and R2R2) was above 80% in all RJ populations, ranging from 82% (Ipn) to 100% (Cgy) (Table 4).

**Table 4:** Genotypic and allelic frequencies of *Aedes aegypti* populations from RJ.

Population	n	SS	SR1	SR2	R1R1	R1R2	R2R2	Allelic frequency			Total R
								S	R1	R2	(R1R1+R1R2+R2R2)
Cgy	46	0	0	0	0	0.07	0.93	0.00	0.04	0.96	1.00
Ibr	44	0	0.05	0.19	0	0.38	0.38	0.02	0.39	0.59	0.96
Igg	44	0	0	0.11	0.09	0.46	0.34	0.04	0.34	0.62	0.91
Ipn	43	0	0	0.07	0.07	0.42	0.44	0.03	0.29	0.68	0.93
Mgr	44	0	0.02	0.09	0.16	0.35	0.38	0.09	0.27	0.64	0.82
Vsr	45	0	0	0.02	0.04	0.27	0.67	0.02	0.16	0.82	0.96

’n’ represents the number of samples evaluated per population. The last column represents the sum of resistant genotypes per population, represented by the aggregate of all resistant genotypes present in that particular population. S here refers to Na_V_S (V1016 + F1534), R1 to *Na*_*V*_*R1* (V1016 + 1534C), and R2 to *Na*_*V*_*R2* (1016I + 1534C)

### Inferring genetic ancestry and population structure

**Fig 6:**
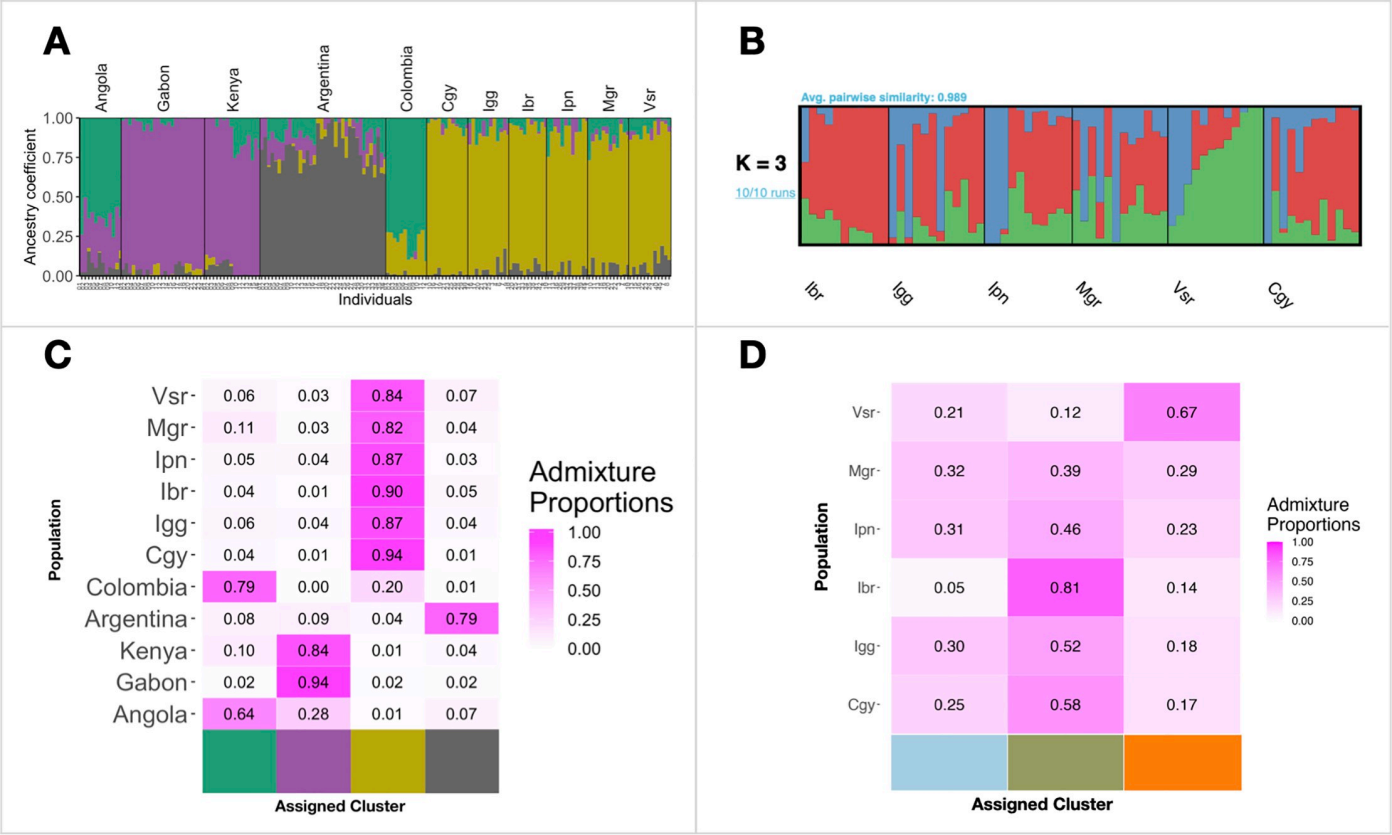
Genetic ancestry of *Aedes aegypti* populations from Rio de Janeiro. A) with a reference panel of the global populations of *Ae*. *aegypti*, estimated using 21,601 SNP loci in LEA with K = 4. Each bar represents one individual. Different colors represent different genetic clusters, and the proportion of a color indicates the probability of an individual assigned to that genetic cluster. We obtained population from other countries described in Kotsakiozi et al. 2018 [90]. Population names and regions are on the x-axis. B) only populations from Rio de Janeiro State with K = 3. C and D. Admixture proportions of each population.

First, we used samples from other countries described previously [44] (S1 Table) to evaluate the RJ *Aedes aegypti* populations’ genetic ancestry. We used 14,095 SNP loci, which passed our filtering thresholds, to infer the genetic ancestry. The best number of clusters was four for the LEA analysis. Similar to what is described by [44] populations from Argentina have ancestry related to African populations (Fig 6A and 6C). Rio de Janeiro populations formed a unique cluster. However, we observe some admixture with mosquitoes from Colombia and Africa as well.

Second, we analyzed the populations from Rio de Janeiro separately. We used 15,321 SNP loci that passed our filtering thresholds to infer their genetic ancestry. The best number of clusters based on the lowest entropy was three. We observed that Ibr, Vsr, and Cgy have the lowest admixture proportions (Figs 6B, 6D and S2). Mgr, Igg, and Ipn have a high degree of admixture and could not be assigned to a specific genetic cluster, indicating low genetic differentiation.

**Fig 7:**
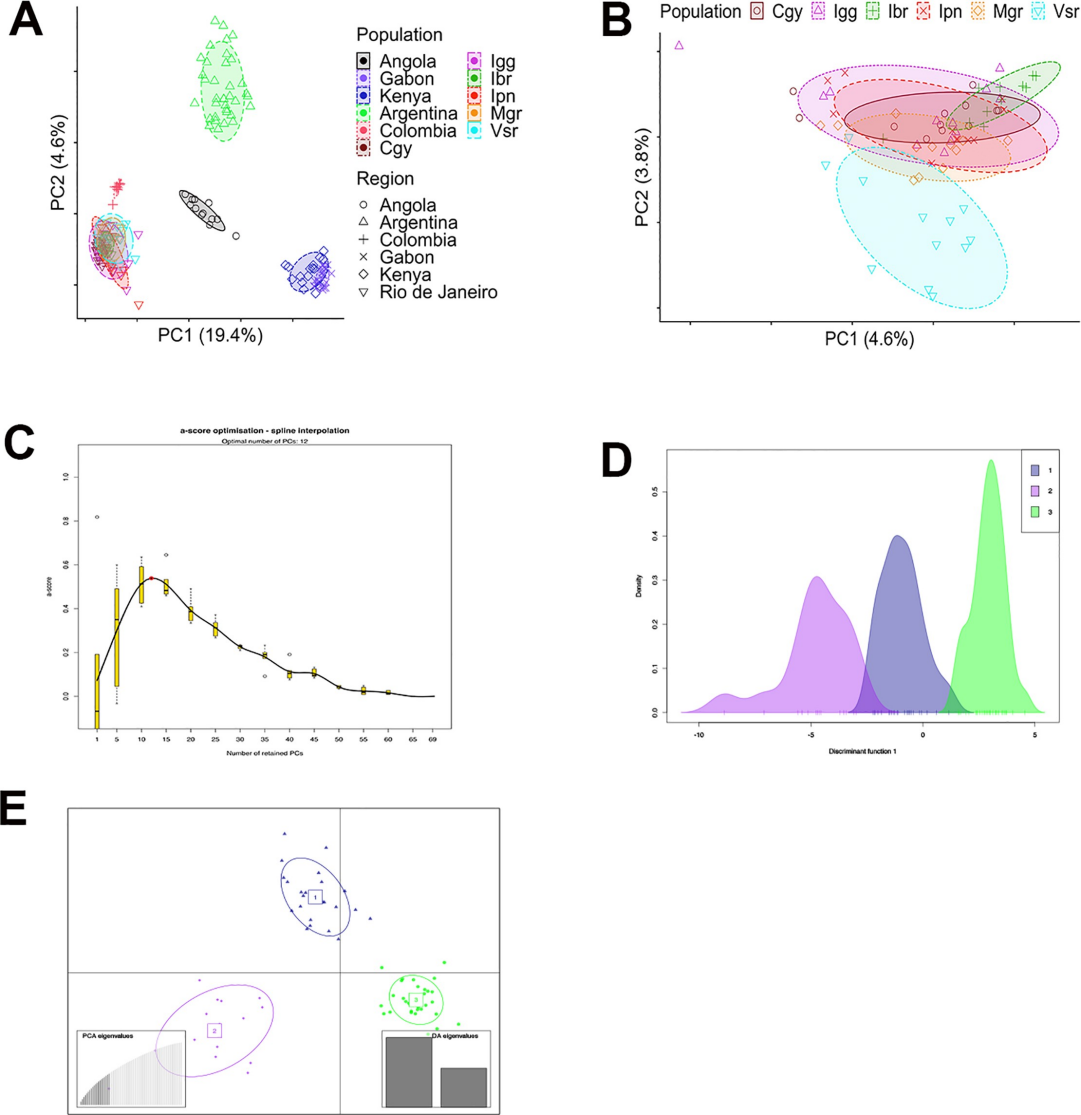
PCA plots showing the first two principal components (PCs). The numbers in the parentheses on the axes indicate the percentage of total variation explained by the PCs. The eclipses represent an 80% confidence level. A. Clustering of populations from Rio de Janeiro State with other populations from different countries. B. Clustering the populations from Rio de Janeiro State only. C. Ideal number of principal components used with DAPC. D. Discriminant function 1 showing three distinct genetic clusters in our data with minimal overlap. E. DAPC for our populations. The graph represents the individuals as dots and the groups as inertia ellipses. A bar plot of eigenvalues for the discriminant analysis (DA eigenvalues) at the inset at the right. The inset bars represent the number of discriminant functions retained in the calculations; we used the first two to generate the plot. On the left, a graph shows how many principal components we used.

The PCA analysis generated similar results when comparing populations from Rio de Janeiro and other countries (Fig 7A). We also performed PCA analysis with only RJ populations (Fig 7B), showing Vsr and Ibr in different clusters, whereas the remaining populations are mixed. The Discriminant Analysis of Principal Components (DAPC) on the allele frequencies showed three clear genetic groups in our data using twelve principal components (Fig 7C–7E). While PCA searches for the direction showing most of the variance in a dataset, the discriminant analysis (DA) maximizes the separation between groups while minimizing group variation. Consequently, PCA may not discriminate groups that DA adequately displays differences.

**Fig 8:**
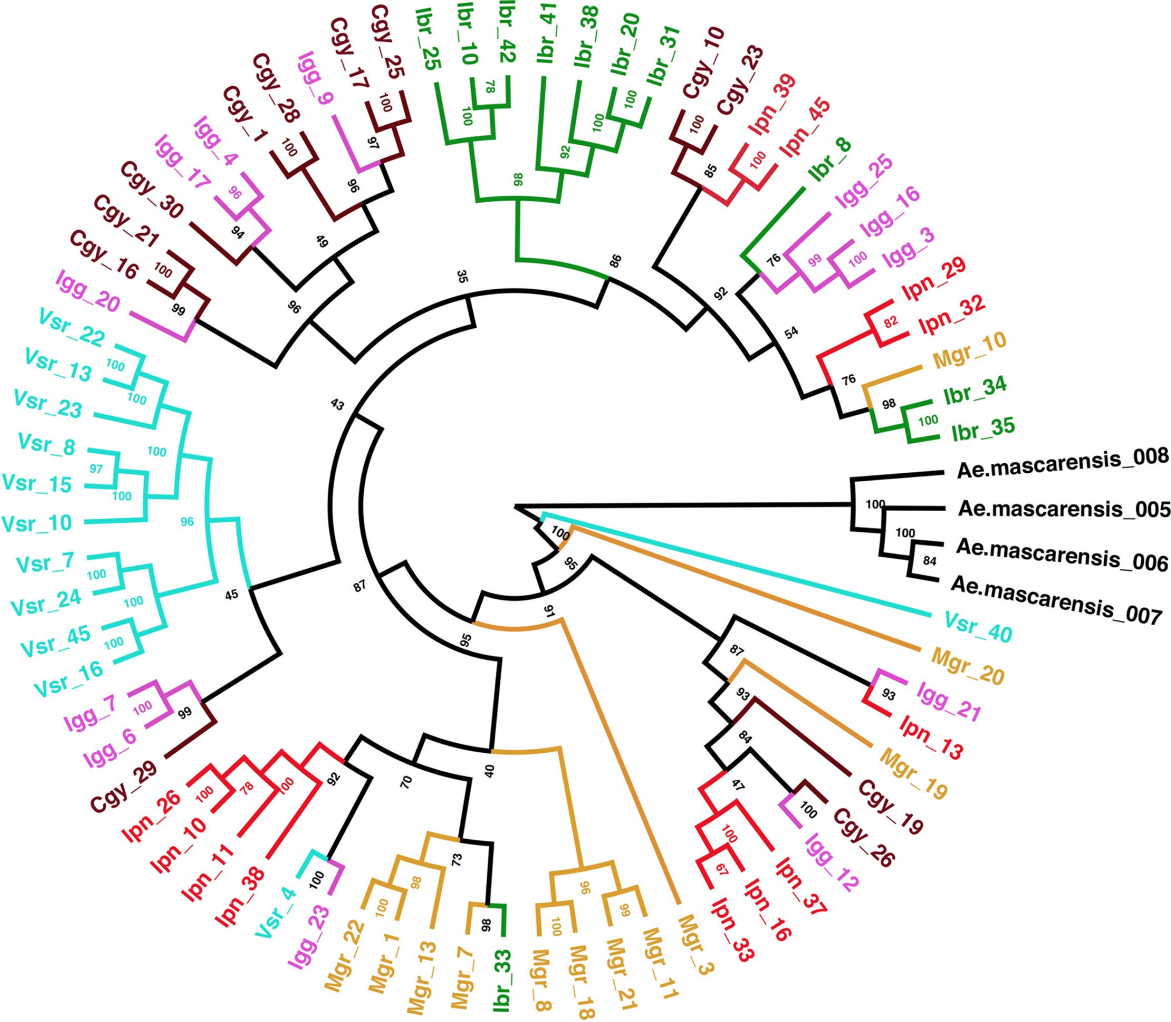
Phylogeny of the Rio de Janeiro mosquitoes generated in IQTree. We developed the tree with 16,596 SNP loci. We used four *Ae*. *mascarensis* samples as the outgroup. The number on each node indicates the support level estimated by 1000 ultrafast bootstraps.

Finally, we constructed a phylogeny with our populations and used *Aedes mascarensis* as an outgroup following [44]. After a new SNP call and filtering, we obtained 16,596 SNP loci for our analysis with IQTree. Our tree supported the results, as mentioned earlier, for Vsr and Ibr unique clustering (Fig 8).

### Detecting heterogeneous differentiation across the genome

**Fig 9:**
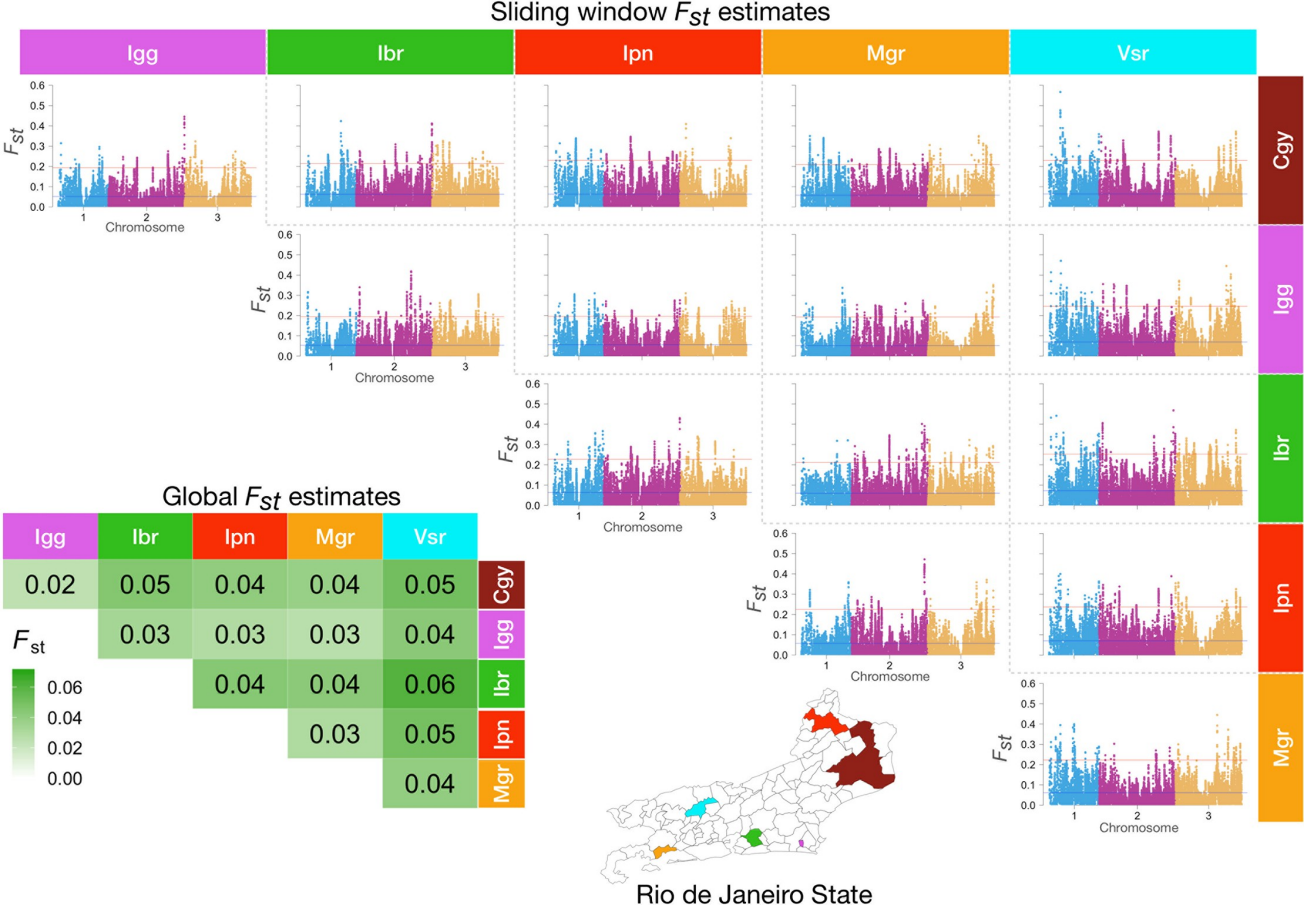
Global estimates of genetic differentiation (*F*_*st*_) among populations of *Aedes aegypti* in Rio de Janeiro (lower left) and sliding window estimates across the genome (upper right).

We estimated the global pairwise *F*_*st*_ values for all populations using 15,321 SNP loci. The overall values are relatively low, but we could observe that mosquitoes from Vsr and Ibr showed the highest genetic differentiation (*F*_*st*_ = 0.06) (Fig 9 and S2 Table). The populations with the lowest genetic differentiation were Igg and Cgy (*F*_*st*_ = 0.02). Since a single *F*_*st*_ estimate may not reveal genetic differentiation in specific genomic regions, we used sliding window analysis to see if we could find such areas. We were able to find sites with higher genetic differentiation even though the global *F*_*st*_ values were low (Fig 9). We could also observe genomic regions with consistent low genetic differentiation across several populations.

### Contemporary migration

We sought to obtain an overall migration trend among RJ localities by detecting first or second-generation mosquito migrants with BA3-SNPs. We found the highest migration estimates from Igg to Cgy (Figs 5 and S1). Igg seemed to be the source and sink of migrants to and from other populations. We did not detect any migrants in Vsr and Ipn.

## Discussion

In Brazil, the 2015 Zika outbreak led to health officials believing that the 2016 Summer Olympic and Paralympic games events, held in Rio de Janeiro, could cause a more significant higher risk of infection of Brazilians, tourists, and athletes [58]. Based on this information, a group of scientists and physicians recommended canceling or postponing the games in Brazil [59]. This was discouraged by the Brazilian government and WHO, because the number of cases dropped [60,61]. Besides, the games were scheduled for August that year, when the density of *Ae*. *aegypti* is usually low. The Brazilian government strengthened vector control measures at the beginning of May with a program named MIRA 360°. Based on WHO protocols of monitoring and targeted actions, the plan used the additional employment of ultrasonic pulses for killing mosquito larvae [62]. According to WHO, among the athletes and visitors, no evidence of Zika infection occurred during the games due to the natural lower density of *Ae*. *aegypti* in August, or because of the intensified vector control measures and self-protection [63]. So far, there is also no evidence of the circulation of arbovirus strains that might have arrived in Brazil during that period. In the following years (2017–2019), although RJ registered a declining number of Zika cases, under 3,000 yearly cases; dengue fluctuated between 10–32 thousand cases, and chikungunya had a high increase from around 10 thousand to more than 85 thousand cases [64]. Adding to this, given the recent outburst of sylvatic yellow fever virus (YFV) in urban centers, there is an urgent need for efficient control strategies of *Ae*. *aegypti* in RJ. This includes monitoring insecticide efficacy [65], and knowledge of genetic differentiation and gene flow dynamics of *Ae*. *aegypti* within RJ.

We used different molecular and population genetic tools and bioassays to characterize populations of mosquitoes collected in cities representing most RJ regions. *Ae*. *aegypti* originated from Africa and spread to the Americas 400 to 500 years ago [66]. The anthropophilic subspecies is *Aedes aegypti aegypti* (Aaa) that breeds in human-made habitats and is present throughout the world [66] and widespread in RJ. The introduction of *Ae*. *aegypti* in the Americas was via Brazil or northern Argentina [7]. Only Argentinian populations have a genetic signature of African populations and are genetically distinct from Brazilian populations [19,67]. One possible reason for this difference may be the eradication program of *Ae*. *aegypti* in Brazil in the 1950s, which was successful until mosquitoes likely from Venezuela and the Caribbean, re-invaded the country [67]. The eradication program in Argentina was not successful [68], and contemporary populations could represent a remnant population that escaped the eradication. Therefore, one of our questions was to see if *Ae*. *aegypti* from RJ is genetically differentiated from other localities and population structure within RJ.

Taking advantage of a recently developed high-density SNP-chip for *Ae*. *aegypti* [38], we evaluated the genetic structure of *Ae*. *aegypti* within RJ populations. Although the chip presents more than 50,000 polymorphic sites, we know it cannot capture all the species’ genetic diversity. After stringent filtering, we were left with approximately 15,000 high-quality SNPs. The previous knowledge about genetic diversity *Ae*. *aegypti* from RJ was limited to populations from the city capital and based upon isoenzymes and microsatellite markers. The level of genetic diversity within the capital limits using isoenzymes was high [20], while microsatellite data showed high admixture levels with populations from other states or regions [18]. These contrasting findings are probably due to the scale on which each study looked at the genetic differentiation. While only comparing samples collected within the 40 km range of Rio de Janeiro city, a higher level of diversity could be observed with isoenzymes [20]. Comparing the same population with others from neighboring states or regions showed a high level of admixture [18]. Such observation could be due to migration caused by the human movement since Rio de Janeiro city is one of Brazil’s major transportation hubs. There is also a fitness advantage of having a resistant insecticide phenotype. Migration and the fitness advantage of carrying resistant alleles may have decreased population structure throughout RJ.

Using about 15,000 biallelic SNP loci, we observed significant genetic differentiation within RJ populations. Population-based analysis, such as PCA and DAPC, indicates three distinct genetic clusters (Fig 7C). However, individual-based analysis, structure, and migration did not show apparent clustering or isolation (Fig 6B). When including similar available data from populations of other countries, RJ populations formed a unique genetic cluster closer to Colombian than Argentinian populations, as observed in the PCA analysis. Previous studies showed that the current populations of *Ae*. *aegypti* in Brazil are originated from re-invasion events, likely from Venezuela, after the eradication efforts in the 1950s [69]. However, in Argentina, the eradication was not completed, as *Ae*. *aegypti* populations from this country show admixture with African populations differently from other South American and Caribbean populations [18,68]. Although we see similar admixture levels between RJ/Africa and Argentina/Africa populations, a previous study using Bayesian inference showed Argentinian populations with an African [18]. As a perspective, when we have available data concerning populations from other Brazilian localities, we will evaluate to what extent populations from RJ similar to others. Our study’s potential knowledge about the vector spatial heterogeneity and data about the vector abundance will be crucial for disease modeling and other vector control programs [70].

Regarding insecticide resistance analyses, RJ populations were resistant to the PY deltamethrin and the OP temephos while susceptible to the IGR pyriproxyfen and the OP malathion. We found the *kdr* resistant alleles at high frequencies. The highest resistance levels to temephos come from *Ae*. *aegypti* populations in Brazil and other Latin American countries, such as French Guiana and the Caribbean [8]. The use of temephos in Brazil dates since 1986 in the metropolitan region of Rio de Janeiro city, following the first reports of dengue cases [2]. Due to resistance to this compound detected in populations throughout the country, temephos was gradually replaced by IGRs since 2008. The last supply of temephos purchased by MoH to RJ was in 2011 [71]. First temephos resistance data in RJ *Ae*. *aegypti* populations came from collections in urban localities around the state capital in 1999, where RR_50_ varied from 3.0 to 7.6 [72]. In the next IR monitoring rounds, between 2001 and 2004, higher resistance ratios were detected, with RR_50_ above 10 in Rio de Janeiro City (13.2 in 2002), São Gonçalo (15.5 in 2001), and Niterói (13.5 in 2001) [73]. In 2006, the Brazilian MoH reduced the resistance ratio threshold from 10 to 3 as a criterion to justify the replacement of temephos with an alternative larvicide [71,74].

A decrease in the resistance levels to temephos occurred in populations from different Brazilian regions due to the substitution of this OP by IGR compounds. For instance, the temephos RR_95_ dropped from 16.3 to 9.8 between 2009 and 2012 in the *Ae*. *aegypti* population from Duque de Caxias in RJ [75]. Among the populations we evaluated, Cgy showed an RR_90_ of 7.8 in 2001 [73], representing a 3-fold reduction in 15 years (RR_90_ of 2.6 in 2016). Ipn registered the highest RRs in RJ populations: RR_95_ equal to 16.8 in 2003, increased to 25.6 in 2011, and decreased to 7.3 in 2016 [71,76]. With the disuse of temephos over the past ten years, susceptibility has expectedly been recovered in Cgy, Ibr, and Igg (RR lower than 3.0). Field-simulated tests are necessary to assess whether temephos would be again a good larvicide option in these localities. On the other hand, high RR to temephos persist in Ipn and Vsr, maybe due to population isolation.

The IGR pyriproxyfen, a juvenile hormone analog, started being used in 2013 against *Ae*. *aegypti* in Brazil. Despite resistance to neurotoxic insecticides in natural populations across the State, the selected mechanisms did not confer any cross-resistance to the IGR in *Ae*. *aegypti* populations from RJ. In our study, all localities were susceptible to pyriproxyfen. As the use of IGRs is recent for mosquito control, few studies reported resistance to these compounds. In one of the few examples, an *Ae*. *aegypti* population from Malaysia showed resistance to the juvenile hormone analog methoprene (RR of 12.7) but low RR (1.4) to pyriproxyfen [77], suggesting a precise resistance mechanism for methoprene. The most recent round of IR monitoring in *Ae*. *aegypti* from Brazil detected resistance to pyriproxyfen (dose-diagnostic assay) in localities from Bahia and Ceará States, in the Northeast of the country [78].

There is evidence of adulticide pyrethroids resistance in RJ *Ae*. *aegypti* populations since the beginning of the 2000s. For instance in 2003, low mortality to cypermethrin was recorded in Cgy (73.3%) and Ipn (54.8%) [79]. Due to continuous reports of pyrethroid resistance throughout the country [80], the MoH decided to replace it with the organophosphate malathion, the only possible alternative recommended by WHO [81], which in RJ started in 2006. Ipn population, for instance, exhibited an RR_90_ equal to 29.7 to deltamethrin later in 2011 [71]. Although pyrethroids’ use in RJ stopped in 2011 [71], we observed that all populations in our study were still resistant to pyrethroids and with high frequencies of *kdr* alleles. Accordingly, in the Ipn population, 48.1% of genotyped mosquitoes presented a *kdr* resistant genotype (R1R1, R1R2, and R2R2) in 2011 [71] and increased to 82% in 2016, indicating persistency in the selection pressure. Previous studies in other parts of Brazil and Mexico show household sprayed insecticides increasing the chance of selecting pyrethroid-resistant *Ae*. *aegypti* populations [82,83]. Besides, in urban centers such as Rio de Janeiro City, private companies are requested by residential condominiums for insecticide applications against the nuisance *Culex quinquefasciatus* mosquitoes, which end up reaching *Ae*. *aegypti* populations. The application is made by fogging or ULV equipment, with pyrethroid based compounds, especially because of their comparatively less irritating smell. These treatments occur several times per week or even daily in some regions during the summer. Therefore, the fact that pyrethroids are no longer deployed by governmental campaigns to control mosquito populations, we still have selection pressure coming from other forms of use of pyrethroids.

In our study, all populations of *Ae*. *aegypti* were utterly susceptible to malathion, even though its use in RJ officially started nine years ago. The Brazilian national insecticide resistance program tested a population from our study, Cgy, and reported it as susceptible in 2018 [78]. Other populations from different cities around the State, the capital, Volta Redonda, and Angra dos Reis, were resistant to malathion. We did not test these populations in our study, and more analyses are necessary to evaluate the extent of the malathion resistance throughout the State [78].

The *kdr* mutations were near fixation in all RJ populations (Fig 5), as most mosquitoes carry both resistance alleles (R1 and R2). We recently demonstrated that there are few *de novo* origins of *kdr* mutations in *Ae*. *aegypti* populations, and therefore, their dispersion is likely due to migration [23]. We can consider this as an adaptative introgression [84,85]. The constant use of pyrethroids in populations with resistant alleles may select insects carrying such alleles, since they have higher fitness in an insecticide-treated environment. Therefore, we expected that the dispersion of a *kdr* allele would contribute to decrease the genetic variability among populations in this scenario. However, we still observe genetic differentiation among the populations studied, with different levels of differentiation across the genome (Fig 9). It means that although we observe a diverse genetic background within RJ, resistance alleles with single origins are present. Similarly, a recent study revealed that new invasions of *Ae*. *aegypti* in localities from the Indo-Pacific are responsible for *kdr* allele dissemination in that region. Distinct populations are well structured, considering their genome-wide variations. However, the SNPs inside or around the voltage-gated sodium channel gene were determined by the individual resistance profile [86]. The constant pyrethroid insecticide pressure seemed to have acted as a vital selection force to drive the kdr alleles’ introgression in the genomic regions that confer resistance, yet without disrupting the overall genome background.

New chemicals have recently been approved by the WHO PQT-VC [87], expecting their use in localities where resistance to multiple compounds is detected. Constant surveillance about the efficacy of old and new compounds is crucial to a well-structured policy of vector control, especially in localities with intense transit of people and cargo inside the country and with nations, as is the case of Rio de Janeiro State. National-wide insecticide resistance monitoring (IRM) programs are an essential part of vector control programs. Still, they generally consider a few mosquito populations from sentinel or strategic localities to be tested due to logistics, personnel and resource limitations. Centralized IRM in big countries like Brazil requires complex logistics and is subjected to discontinuities, letting some regions out of the analyses and evaluations less frequently than necessary. States and provinces must prepare to conduct IRM, for example, how it is currently done in São Paulo State [39]. National vector control programs should invest in technical training and infrastructure in states or provinces to improve their IRM data, based on continuous data collection in most of their cities. Besides, the knowledge of the genetic structure and gene flow dynamics among the natural populations can be used as a surveillance tool, with high potential to guide vector control interventions. Investments in sanitary infrastructure, scientific development, education, and community engagement have to improve continuously, or none of the strategies will succeed to currently known and new emergent arboviruses transmission.

## Conclusions and future directions

Selection pressure and fitness cost play an essential role in recovering any vector population’s susceptible status. We observed that populations from RJ are recovering susceptibility to larvicide OP temephos. Populations are susceptible to larvicide IGR pyriproxyfen and adulticide OP malathion. Resistance to deltamethrin continues in all populations, with high frequencies of alleles related to knockdown resistance. Continuing IR monitoring is required to see if populations obtain complete susceptibility to temephos and keep a check on resistance to malathion and pyriproxyfen.

It is uncertain if there is any impact of resistance levels on the vectorial capacity of *Ae*. *aegypti* populations across RJ. It will be essential to evaluate each genetic cluster’s vector capacity to guide the vector control programs towards more competent populations. The *Ae*. *aegypti* SNP-chip proved to be a useful tool for determining the genetic structure and providing critical clues about the gene flow among mosquitoes collected even in proximate cities. It will also be vital to have temporal data from all populations to evaluate the migration within the State and if there are changes in the genetic structure between the dry and wet seasons. Migration and hybridization might play an essential role in spreading and maintaining the insecticide resistance alleles. Temporal surveys of IR estimates and the corresponding molecular markers, such as *kdr*, are crucial to track the rise or introduction of other resistance alleles in the State. If new resistant haplotypes appear, vector control programs will be necessary to know where and how fast they will spread. Finally, knowledge of genetic diversity and gene flow will be needed to optimize new vector control tools such as *Wolbachia* carrying mosquitoes [88] and RIDL transgenic mosquitoes [89].

## Supporting information

S1 Text Official inhabitant census and incidence of arboviruses in Rio de Janeiro State, Brazil. **A**. Official census number of inhabitants per year in Rio de Janeiro State. **B**. Dengue cases registered in the cities of the Rio de Janeiro State. **C**. Percentual of Dengue cases of each city proportional to total cases in the State. **D**. Percentual of Dengue cases of each city proportional to total cases in the State. **E**. Incidence of Dengue cases (%). **F**. Chikungunya cases in the cities of the Rio de Janeiro State. **G**. Percentual of Chikungunya cases of each city proportional to total cases in the State. **H**. Incidence of Chikungunya cases (%). **I**. Zika cases registered in the cities of the Rio de Janeiro State. **J** Percentage of Zika cases registered in the cities of the Rio de Janeiro State. **K**. Incidence of Zika cases (%).
(PDF)

S1 Table Population information for the *Ae*. *aegypti* samples used in this study from Kotsakiozi et al (2018)*. (PDF)

S2 Table Pairwise weighted Weir & Cockerham *F*_*st*_ estimates for all populations from the R package StTAMPP. We used 100 bootstraps to calculate *p*-values and confidence intervals (95%).
(PDF)

S1 Fig Contemporary migration estimates of *Aedes aegypti* in Rio de Janeiro State and frequency of the *kdr* alleles. (TIF)

S2 Fig Genetic ancestry of *Aedes aegypti* populations from Rio de Janeiro. (TIF)
