# Supplementary material for: Insecticide resistance and genetic structure of Aedes aegypti populations from Rio de Janeiro State, Brazil
Source: PLoS Negl Trop Dis. 2021 Feb 16;15(2):e0008492. doi: 10.1371/journal.pntd.0008492 (PMC7909666; doi:10.1371/journal.pntd.0008492)
Supplement: S1 Text — A. Official census number of inhabitants per year in Rio de Janeiro State. B. Dengue cases registered in the cities of the Rio de Janeiro State. C. Percentual of Dengue cases of each city proportional to total cases in the State. D. Percentual of Dengue cases of each city proportional to total cases in the State. E. Incidence of Dengue cases (%). F. Chikungunya cases in the cities of the Rio de Janeiro State. G. Percentual of Chikungunya cases of each city proportional to total cases in the State. H. Incidence of Chikungunya cases (%). I. Zika cases registered in the cities of the Rio de Janeiro State. J Percentage of Zika cases registered in the cities of the Rio de Janeiro State. K. Incidence of Zika cases (%). (PDF) [file pntd.0008492.s001.pdf]

Insecticide resistance and genetic structure of *Aedes aegypti* populations from Rio de Janeiro State, Brazil.

Rafi Ur Rahman, Luciano Veiga Cosme, Monique Melo Costa, Luana Carrara, José Bento Pereira Lima, Ademir Jesus Martins

Support Information

S1 Text. Official inhabitant census and incidence of arboviruses in Rio de Janeiro State, Brazil.

Table A. Official census number of inhabitants per year in Rio de Janeiro State.

Localities	2015	2016	2017	2018
Rio de Janeiro (capital)	6,476,631	6,498,837	6,520,266	6,688,927
Itaboraí	229,007	230,786	232,394	238,695
Itaperuna	99,021	99,504	99,997	102,626
Campos dos Goytacazes	483,970	487,186	460,288	503,429
Iguaba Grande	25,901	26,430	26,936	27,762
Vassouras	35,432	35,622	35,768	36,702
Mangaratiba	40,779	41,557	232,394	43,689

Source: IBGE

Table B. Dengue cases registered in the cities of the Rio de Janeiro State.

Localities	2015	2016	2017	2018
Rio de Janeiro (capital)	17,844	25,650	3,622	5,302
Itaboraí	743	761	653	2,731
Itaperuna	2,729	3,768	212	289
Campos dos Goytacazes	4,613	1,443	59	147
Iguaba Grande	28	10	4	28
Vassouras	768	521	74	40
Mangaratiba	176	32	7	4
Rio de Janeiro State	73,167	82,280	10,806	14,763

Source: SINAN, GDTVZ, SES/RJ data revised on January 29th, 2019.

Table C. Percentual of Dengue cases of each city proportional to total cases in the State.

Localities	2015	2016	2017	2018
Rio de Janeiro (capital)	17,844	25,650	3,622	5,302
Itaboraí	743	761	653	2,731
Itaperuna	2,729	3,768	212	289
Campos dos Goytacazes	4,613	1,443	59	147
Iguaba Grande	28	10	4	28
Vassouras	768	521	74	40
Mangaratiba	176	32	7	4
Rio de Janeiro State	73,167	82,280	10,806	14,763

Table D. Percentual of Dengue cases of each city proportional to total cases in the State.

Localities	2015	2016	2017	2018
Rio de Janeiro (capital)	24.4	31.2	33.5	35.9
Itaboraí	1.0	0.9	6.0	18.5
Itaperuna	3.7	4.6	2.0	2.0
Campos dos Goytacazes	6.3	1.8	0.5	1.0
Iguaba Grande	0.0	0.0	0.0	0.2
Vassouras	1.0	0.6	0.7	0.3
Mangaratiba	0.2	0.0	0.1	0.0
Rio de Janeiro State	100	100	100	100

Table E. Incidence of Dengue cases (%).

Localities	2015	2016	2017	2018
Rio de Janeiro (capital)	2.8	3.9	0.6	0.8
Itaboraí	3.2	3.3	2.8	11.4
Itaperuna	27.6	37.9	2.1	2.8
Campos dos Goytacazes	9.5	3.0	0.1	0.3
Iguaba Grande	1.1	0.4	0.1	1.0
Vassouras	21.7	14.6	2.1	1.1
Mangaratiba	4.3	0.8	0.0	0.1
Rio de Janeiro State	4.4	4.9	0.6	0.9

According to Brazilian MoH, values >0.3 indicate an epidemic condition.

Table F. Chikungunya cases in the cities of the Rio de Janeiro State.

Localities	2015	2016	2017	2018
Rio de Janeiro (capital)	18	13,843	3,571	9,774
Itaboraí	-	9	622	6,212
Itaperuna	-	-	211	292
Campos dos Goytacazes	1	1	57	7,304
Iguaba Grande	-	17	4	104
Vassouras	-	-	71	3
Mangaratiba	-	2	7	22
Rio de Janeiro State	67	15,414	10,595	39,082

Source: SINAN, GDTVZ, SES/RJ data revised on January 29th, 2019.

Table G. Percentual of Chikungunya cases of each city proportional to total cases in the State.

Localities	2015	2016	2017	2018
Rio de Janeiro (capital)	0.0	16.8	33.0	66.2
Itaboraí	-	0.0	5.8	42.1
Itaperuna	-	-	2.0	2.0
Campos dos Goytacazes	0.0	0.0	0.5	49.5
Iguaba Grande	-	0.0	0.0	0.7
Vassouras	-	-	0.7	0.0
Mangaratiba	-	0.0	0.1	0.1
Rio de Janeiro State	0	19	98	265

Table H. Incidence of Chikungunya cases (%).

Localities	2015	2016	2017	2018
Rio de Janeiro (capital)	0.0	2.1	0.5	1.5
Itaboraí	-	0.0	2.7	26.0
Itaperuna	-	-	2.1	2.8
Campos dos Goytacazes	0.0	0.0	0.1	14.5
Iguaba Grande	-	0.6	0.1	3.7
Vassouras	-	-	2.0	0.1
Mangaratiba	-	0.0	0.0	0.5
Rio de Janeiro State	0.0	0.9	0.6	2.3

Table I. Zika cases registered in the cities of the Rio de Janeiro State.

Localities	2015	2016	2017	2018
Rio de Janeiro (capital)	7,262	32,146	649	541
Itaboraí	45	4,128	83	84
Itaperuna	1	252	5	-
Campos dos Goytacazes	159	4,657	127	1
Iguaba Grande	-	2	-	1
Vassouras	-	4	-	-
Mangaratiba	1	181	1	-
Rio de Janeiro State	10,405	72,359	2,597	2,339

Source: SINAN, GDTVZ, SES/RJ data revised on January 29th, 2019.

Table J Percentage of Zika cases registered in the cities of the Rio de Janeiro State.

Localities	2015	2016	2017	2018
Rio de Janeiro (capital)	9.9	39.1	6.0	3.7
Itaboraí	0.1	5.0	0.8	0.6
Itaperuna	0.0	0.3	0.0	-
Campos dos Goytacazes	0.2	5.7	1.2	0.0
Iguaba Grande	-	0.0	-	0.0
Vassouras	-	0.0	-	-
Mangaratiba	0.0	0.2	0.0	-
Rio de Janeiro State	14	88	24	16

Table K. Incidence of Zika cases (%).

Localities	2015	2016	2017	2018
Rio de Janeiro (capital)	1.1	4.9	0.1	0.1
Itaboraí	0.2	17.9	0.4	0.4
Itaperuna	0.0	2.5	0.1	-
Campos dos Goytacazes	0.3	9.6	0.3	0.0
Iguaba Grande	-	0.1	-	0.0
Vassouras	-	0.1	-	-
Mangaratiba	0.0	4.4	0.0	-
Rio de Janeiro State	0.6	4.3	0.2	0.1
