# Supplementary material for: Insecticide resistance and genetic structure of Aedes aegypti populations from Rio de Janeiro State, Brazil
Source: PLoS Negl Trop Dis. 2021 Feb 16;15(2):e0008492. doi: 10.1371/journal.pntd.0008492 (PMC7909666; doi:10.1371/journal.pntd.0008492)
Supplement: S1 Table — (PDF) [file pntd.0008492.s002.pdf]

Insecticide resistance and genetic structure of *Aedes aegypti* populations from Rio de Janeiro State, Brazil.

Rafi Ur Rahman, Luciano Veiga Cosme, Monique Melo Costa, Luana Carrara, José Bento Pereira Lima, Ademir Jesus Martins

Support Information

S1 Table. Population information for the *Ae. aegypti* samples used in this study from Kotsakiozi et al (2018)*

Locality [map code]	Region	Latitude	Longitude	N
Colombia	South America	3.43894	-76.516	12
Kenya	Africa	-3.93194	39.5961	8
Gabon	Africa	−0.37896	11.5274	12
Angola	Africa	−9.76667	14.26667	12

* Kotsakiozi P, Evans BR, Gloria-Soria A, Kamgang B, Mayanja M, Lutwama J, et al. Population structure of a vector of human diseases: *Aedes aegypti* in its ancestral range, Africa. *Ecol Evol*. 2018;8(16):7835-48. Epub 2018/09/27. doi: 10.1002/ece3.4278.
