# Supplementary material for: Insecticide resistance and genetic structure of Aedes aegypti populations from Rio de Janeiro State, Brazil
Source: PLoS Negl Trop Dis. 2021 Feb 16;15(2):e0008492. doi: 10.1371/journal.pntd.0008492 (PMC7909666; doi:10.1371/journal.pntd.0008492)
Supplement: S2 Table — We used 100 bootstraps to calculate p-values and confidence intervals (95%). (PDF) [file pntd.0008492.s003.pdf]

Insecticide resistance and genetic structure of *Aedes aegypti* populations from Rio de Janeiro State, Brazil.

Rafi Ur Rahman, Luciano Veiga Cosme, Monique Melo Costa, Luana Carrara, José Bento Pereira Lima, Ademir Jesus Martins

Support Information

S2 Table. Pairwise weighted Weir & Cockerham F_{st} estimates for all populations from the R package StTAMPP. We used 100 bootstraps to calculate *p*-values and confidence intervals (95%).

Population1	Population2	F_{st}	Lower bound CI limit	Upper bound CI limit	*p*-value*
Cgy	Igg	0.02	0.02	0.03	0.001
Cgy	Ibr	0.05	0.04	0.05	0.001
Cgy	Ipn	0.04	0.04	0.04	0.001
Cgy	Mgr	0.04	0.04	0.04	0.001
Cgy	Vsr	0.05	0.05	0.05	0.001
Igg	Ibr	0.03	0.03	0.03	0.001
Igg	Ipn	0.03	0.03	0.03	0.001
Igg	Mgr	0.03	0.02	0.03	0.001
Igg	Vsr	0.04	0.04	0.04	0.001
Ibr	Ipn	0.04	0.04	0.05	0.001
Ibr	Mgr	0.04	0.04	0.04	0.001
Ibr	Vsr	0.06	0.06	0.06	0.001
Ipn	Mgr	0.03	0.03	0.03	0.001
Ipn	Vsr	0.05	0.05	0.05	0.001
Mgr	Vsr	0.04	0.04	0.04	0.001

**p*-values smaller than 0.001
